# Synergistic Effect of Chitosan and Selenium Nanoparticles on Biodegradation and Antibacterial Properties of Collagenous Scaffolds Designed for Infected Burn Wounds

**DOI:** 10.3390/nano10101971

**Published:** 2020-10-05

**Authors:** Jana Dorazilová, Johana Muchová, Kristýna Šmerková, Silvia Kočiová, Pavel Diviš, Pavel Kopel, Radek Veselý, Veronika Pavliňáková, Vojtěch Adam, Lucy Vojtová

**Affiliations:** 1 CEITEC—Central European Institute of Technology, Brno University of Technology, Purkyňova 656/123, 612 00 Brno, Czech Republic; jana.dorazilova@ceitec.vutbr.cz (J.D.); johana.babrnakova@ceitec.vutbr.cz (J.M.); kristyna.smerkova@mendelu.cz (K.Š.); xkociova@mendelu.cz (S.K.); paulko@centrum.cz (P.K.); veronika.pavlinakova@ceitec.vutbr.cz (V.P.); vojtech.adam@mendelu.cz (V.A.); 2 Department of Chemistry and Biochemistry, Faculty of AgriSciences, Mendel University in Brno, Zemědělská 1, 613 00 Brno, Czech Republic; 3 Faculty of Chemistry, Brno University of Technology, Purkyňova 118, 612 00 Brno, Czech Republic; divis@fch.vut.cz; 4 Department of Inorganic Chemistry, Faculty of Science, Palacky University, 17. Listopadu 12, 771 46 Olomouc, Czech Republic; 5 Department of Traumatology at the Medical Faculty, Masaryk University and Trauma Hospital of Brno, Ponavka 6, 662 50 Brno, Czech Republic; ves.radek@seznam.cz

**Keywords:** tissue engineering, drug release, freeze-drying, collagen, chitosan, selenium nanoparticles, infected burn injuries, bacteria, *Staphylococcus*

## Abstract

A highly porous scaffold is a desirable outcome in the field of tissue engineering. The porous structure mediates water-retaining properties that ensure good nutrient transportation as well as creates a suitable environment for cells. In this study, porous antibacterial collagenous scaffolds containing chitosan and selenium nanoparticles (SeNPs) as antibacterial agents were studied. The addition of antibacterial agents increased the application potential of the material for infected and chronic wounds. The morphology, swelling, biodegradation, and antibacterial activity of collagen-based scaffolds were characterized systematically to investigate the overall impact of the antibacterial additives. The additives visibly influenced the morphology, water-retaining properties as well as the stability of the materials in the presence of collagenase enzymes. Even at concentrations as low as 5 ppm of SeNPs, modified polymeric scaffolds showed considerable inhibition activity towards Gram-positive bacterial strains such as *Staphylococcus aureus* and methicillin-resistant *Staphylococcus aureus* and *Staphylococcus epidermidis* in a dose-dependent manner.

## 1. Introduction

The skin is the largest organ of the human body, covering on average an area of 1.8 m^2^ with the main functions of protection, respiration, and homeostasis maintenance [1]. Unlike other organs, the skin is in long-term direct contact with the surrounding environment and is easily injured. Minor skin wounds follow the healing process of homeostasis, inflammation, proliferation, and matrix deposition with the remodeling of the tissue as the final result [2]. However, severely infected wounds larger than 2 cm in diameter follow a slower and more complicated repair process and often fail closure [3,4,5]. Opportunistic skin bacteria from the genus *Staphylococcus* are a common source of infection, namely, *Staphylococcus aureus* or *Staphylococcus epidermidis* [6,7]. Lately, overuse of antibiotics has led to the formation of a strain resistant to β-lactam-derived antibiotics, so-called methicillin-resistant *Staphylococcus aureus* (MRSA) [8]. Proper antibacterial wound dressing can support healing of chronic wounds. Here, tissue engineering (TE) could step-up production of an antibacterial material that could provide the necessary healing support and antimicrobial environment for complex wound management [9,10,11,12].

The ideal regenerative material should resemble the regenerated tissue both in physical structure and in chemical composition [13]. Collagen is the most abundant protein within the extracellular matrix (EMC)—a complex mixture of proteins and proteoglycans arranged in a three-dimensional structure [14]. As a key part of the ECM, collagen plays a crucial part in the induction of tissue regeneration as a mediator of cell communication and a provider of structural reinforcement [15,16]. Widespread as it is, collagen is easily obtainable and processable for applications in TE and can be fabricated into injectable gels, films, meshes, and porous scaffolds [17,18,19,20]. Porous scaffolds remain the most versatile applicable structure in TE, since it closely resembles the vital environment for cells [21,22,23,24]. Despite its unique properties, collagen alone exhibits neglible antimicrobial activity and requires modification to unlock its potential as cellular support in complex wounds [25].

Chitosan is a derivative of polysaccharide chitin (2-(acetylamino)-2-deoxy-D-glucose) produced via enzymatic or chemical deacetylation [26]. The formed polycationic character of protonated amino groups gives chitosan its unique properties in the physiological environment [27,28]. Positively charged chitosan molecules can disrupt negatively charged bacterial cell walls via electrostatic forces [29]. In general, the application of chitosan as an additive in the porous collagen matrix can positively improve both healing and antibacterial properties of the material since, above all, chitosan was reported to have mucoadhesive and hemostatic properties [30,31].

As a part of selenoproteins, selenium is an important biogenic element ensuring proper cellular and metabolic functioning [32]. Crucial properties that make SeNPs suitable agents for medical applications are their antimicrobial, antioxidant, and anticancer properties [33]. A large group of microorganisms are sensitive to SeNPs, consisting of Gram-positive (*Staphylococcus aureus* and MRSA [34,35]) and Gram-negative bacteria (*Pseudomonas aeruginosa* [35,36,37], *Proteus mirabilis* [36], *Acinetobacter baumannii* [37]) as well as yeast (*Candida albicans* [38]) and fungus (*Trichophyton rubrum* [39]).

In this paper, we prepared porous, collagen-based scaffolds modified with chitosan and biopolymer-stabilized SeNPs to enhance the scaffold’s antibacterial properties. The morphology, swelling, biodegradation, and antibacterial activity of collagen-based scaffolds were characterized systematically to investigate the overall effect of the antibacterial additives. We aimed to present a comprehensive study of the impact of antibacterial additives on collagen-based materials and promote the application of nanoparticles in tissue engineering.

## 2. Materials and Methods 

### 2.1. Materials

Bovine collagen type I 100% lyophilized foam was provided by Collado (Brno, Czech Republic). N-(-3-Dimethylaminopropyl)-N’-ethyl carbodiimide hydrochloride (EDC), N-hydroxysuccinimide (NHS), chitosan from shrimp shells, carboxymethyl cellulose sodium salt, acetic acid, Na_2_SeO_3_·5H_2_O, mercaptopropionic acid, NaOH, sodium phosphate dibasic, collagenase from *Clostridium histolyticum* ≥ 125 CDU/mg were purchased from Sigma–Aldrich (Darmstadt, Germany). Ethanol 96% a.r. and ethanol absolute a.r. were obtained from PENTA (Prague, Czech Republic), and chemicals for phosphate-buffered saline (PBS) preparation a.r. were purchased from Lach–Ner (Neratovice, Czech Republic). Ultrapure water type II was purified using a Millipore filtration system according to ISO 3696. All used reagents and solvents were of analytical grade and used as received.

### 2.2. Methods

#### 2.2.1. Preparation of Both Collagen and Collagen–Chitosan Scaffolds 

Collagen suspensions of 0.5% (*w*/*v*) concentration were prepared by disintegrating 100% freeze-dried bovine collagen in ultrapure water. The chosen concentration and cross-linking method were optimized in our previous work [40]. Briefly, disintegrated collagenous suspensions were poured into molds with a radius of 0.5 cm and a height of 1.5 cm. The porous structure was achieved by a freeze-drying procedure (lyophilizator Martin Christ EPSILON 2–10, Osterode am Harz, Germany). All collagenous materials were structurally stabilized by chemical cross-linking via the EDC/NHS system. The chemical cross-linking reaction was terminated using 0.1 M Na_2_HPO_4_, and all used reagents were rinsed away by ultrapure water. After cross-linking treatment, stabilized porous materials were freeze-dried again achieving dried collagen scaffolds (C). Collagen–chitosan scaffolds (CCH) were prepared in a similar manner. Collagen–chitosan suspensions with a total 1% (*w*/*v*) concentration of biopolymers were prepared by disintegration of 100% freeze-dried bovine collagen in ultrapure water with the addition of suspended chitosan to create a mixture of 0.5% (*w*/*v*) collagen and 0.5% (*w*/*v*) chitosan. Afterward, the preparation followed the same protocol as the abovementioned preparation of pure C scaffolds.

#### 2.2.2. Preparation of Collagen and Collagen–Chitosan Scaffolds with SeNPs

Two types of SeNPs differing in biopolymer substance, in which they were anchored, were used in this work. Both types of SeNPs were synthesized according to the procedure published in our previous studies [41,42]. The SeNPs with carboxymethyl cellulose (SeCE) were prepared by the reduction of Na_2_SeO_3_ with mercaptopropionic acid in the presence of carboxymethyl cellulose. The SeNP’s sizes differed from 50 to 300 nm and with selenium contents of approximately 278 ppm. The SeNPs with chitosan (SeCH) were prepared by a similar process of Na_2_SeO_3_ reduction by mercaptopropionic acid in the presence of chitosan. The SeNP’s sizes differed from 55–500 nm with selenium contents of approximately 663 ppm. The application of SeNPs on biopolymeric matrices was achieved as followed. Diluted suspensions of SeNPs with concentrations of 1, 5, and 10 ppm were poured onto the scaffolds after eliminating cross-linking agents. The SeNP-covered scaffolds were freeze-dried afterward. 

Table 1 summarizes the variety of prepared scaffolds. Two biopolymeric matrices—C and CCH—were investigated in this work in a pure state or further modified by one type of synthesized SeNPs—SeCH and SeCE. For physicochemical analysis, SeNPs with a concentration of 5 ppm were used as a midpoint concentration derived from antibacterial testing, where the range of concentrations 1, 5, and 10 ppm was used.

#### 2.2.3. Microstructure Imaging and Evaluation 

The morphology of pure and SeNP-modified (c = 5 ppm) C and CCH scaffolds were investigated by scanning electron microscopy (SEM, MIRA 3, Tescan, Brno, Czech Republic). After 10 min of freezing in liquid nitrogen, freeze-dried porous scaffolds were sliced to obtain a narrow transversal cut for SEM imaging with 10.0 kV acceleration voltage. Images were reconstructed from the detection of secondary electrons and the detection of dispersive X-ray energy. Images were acquired at 175, 250, 274, and 25,000× magnification. Pore sizes were evaluated using ImageJ (National Institutes of Health, Bethesda, MD, USA).

Porosity measurements were carried out using a gravimetrical liquid displacement method. Absolute ethanol was used as a probe which can penetrate the biopolymeric microstructure of the scaffold without swelling. Briefly, pure and SeNP-modified (c = 5 ppm) C and CCH scaffolds with similar weights were immersed in 10 mL of absolute ethanol and removed after 5 min of ethanol adsorption. Porosity was calculated using Equation (1), where *V_e_* stands for the volume of ethanol in the structure calculated from the weight difference of dry and wet scaffolds (SI-234A, DENVER INSTRUMENT, Bohemia, NY, USA) and an ethanol density of 0.79 g.cm^−3^. The variable *V_d_* represents the volume of the scaffold skeleton calculated from the dimensions measured by caliper.

(1) $$\mathrm{Porosity}\;\left(\%\right)=\frac{V_{e}}{V_{d}}\cdot 100$$

Graphs were created in OriginPro2020b (Originlab, Northampton, MA, USA). Each measurement was repeated three times to be expressed with the appropriate standard deviation.

#### 2.2.4. Investigation of Collagen Secondary Structure

Fourier transformed infrared spectroscopy with attenuated total reflectance (ATR-FTIR, Vertex 70/70v, Bruker, Billerica, MA, USA) was performed to evaluate the intact collagen secondary structure. After 10 min of freezing in liquid nitrogen, pure and SeNP-modified (c = 5 ppm) scaffolds were sliced into thin slices (2 mm) using a scalpel and carefully placed on a diamond ATR crystal. The ATR-FTIR spectra were measured in an evacuated condition in a mid-infrared spectral range of 4500–800 cm^−1^, averaging 32 scans with a spectral resolution of 4 cm^−1^. The spectra were corrected in the 4500–800 cm^−1^ spectra region using a rubber band correction algorithm and normalized using min–max normalization (OPUS software, Bruker, Billerica, MA, USA). The triple-helical ratio was calculated according to Reference [43] and Equation (2), where *A_amide III_* and *A_pyrrolidine_* correspond to the absorbances of Amide III bands and pyrrolidine bands.

(2) $$\mathrm{Triple}-\mathrm{helix}\;\mathrm{ratio}\;(-)\;=\;\frac{A_{\mathrm{amide}\;\mathrm{III}}}{A_{\mathrm{pyrrolidine}}}$$

Reciprocal second derivatives of the spectra were computed in the software OriginPro2020b (Originlab, Northampton, MA, USA) and smoothed in the same software by applying the Savitzky–Golay method with nine smoothing points.

#### 2.2.5. Swelling and Water Absorption Studies

Swelling of pure and SeNP-modified (c = 5 ppm) C and CCH scaffolds was observed over time using a gravimetric method. Briefly, dry collagenous scaffolds of similar weight were weighed and placed in vials with 10 mL of ultrapure water. At regular time intervals (1, 3, 5, 10, 15, 20, 30, 45, 60, 150, and 180 min), swelled scaffolds were removed from the vials and weighed on the analytical balances (SI-234A, DENVER INSTRUMENT, Bohemia, NY, USA). Swelling behavior was evaluated through swelling ratios (3) and water content (4).

(3) $$\mathrm{Swelling}\;\mathrm{ratio}\;(-)\;=\frac{w_{d}}{w_{w}}$$

(4) $$\mathrm{Water}\;\mathrm{content}\;(\%)\;=\;\frac{w_{w}-w_{d}}{w_{w}}\;\cdot \;100$$

where *w_w_* is the weight of the wet scaffolds and *w_d_* is the weight of the dry scaffolds. Graphs were created in OriginPro2020b (Originlab, Northampton, MA, USA). Each measurement was repeated three times to be expressed with the appropriate standard deviation.

#### 2.2.6. Degradation in a Simulated Physiological Environment

Evaluation of the enzymatic degradation in simulated physiological conditions was carried out in the collagenase (c = 2.2 mg·L^−1^) solution diluted in 0.01 M PBS. The concentration of collagenase was chosen according to Reference [44]. Pure and SeNP-modified (c = 5 ppm) C and CCH scaffolds were left to swell in PBS without enzymes for an hour. Afterward, porous materials were weighed to obtain *w*_60_ (i.e., the weight of the swelled materials after 60 min when the absorption equilibrium was achieved). After immersion of the materials in PBS solution with enzymes, the materials were removed at regular time intervals (i.e., 2, 4, 8, 24, 48, 72, and 144 h) and weighed to obtain *w_H_* (i.e., the weight of the wet materials at appropriate time intervals). The enzymatic degradation was evaluated using Equation (5):

(5) $$\mathrm{Degradation}\;(\%)\;=\;100\;-\;\frac{{(w}_{H}\;\cdot \;100)}{w_{60}}$$

Graphs were created in OriginPro2020b (Originlab, Northampton, MA, USA). Each measurement was repeated three times to be expressed with the appropriate standard deviation.

#### 2.2.7. Release Kinetics of SeNPs

The release of SeNPs from SeNP-modified (c = 5 ppm) C and CCH scaffolds was studied in ultrapure water at 37 °C. Each scaffold was immersed in 25 mL of ultrapure water. At regular time intervals (30 min, 1, 4, 8, 24, 48 h), 3.5 mL of solution with released SeNPs were removed for further analysis. Determination of the number of released SeNPs was achieved using the optical emission spectrometer with inductively coupled plasma (ICP-OES, Ultima2, Horiba, Kisshoin, Minami-ku Kyoto, Japan) and evaluated using a five-point calibration curve constructed from SeNP solutions with known concentrations. Graphs were created in OriginPro2020b (Originlab, Northampton, MA, USA). Each measurement was repeated three times to be expressed with the appropriate standard deviation.

#### 2.2.8. Antibacterial Activity Evaluation 

The antibacterial properties of pure and SeNP-modified (c = 1, 5, 10 ppm) C and CCH scaffolds were tested on different bacterial strains from the Czech Collection of Microorganisms (Brno, Czech Republic), which represented by both Gram-positive (*S. aureus* ATCC 29213 and MRSA CCM 7110) and Gram-negative (*Escherichia coli* ATCC 25922) bacteria. For better evaluation of antibacterial efficacy in clinical practice, the clinical bacteria isolated from patient’s wounds were also used. A collection of swabs from infected wounds of patients (Trauma Hospital of Brno, Czech Republic) and the subsequent bacteria cultivation was described in a study by Hegerova et al. [42]. The ethics committee of the Trauma Hospital of Brno, Czech Republic, approved this study according to Act no. 378/2007 collection of laws. The collection took place at the Trauma Hospital of Brno. Swabs were collected from infected wounds of patients and cultivated as mentioned above. The two types of *Staphylococcus*, *S. aureus* and *S. epidermidis*, which are commonly found on human skin and likewise to colonize wounds, were treated. Similar to the bacteria from the Czech Collection, the sensitive and resistant strains were tested. Bacterial cultures were cultivated on blood agar plates overnight at 37 °C.

Disk Diffusion Method. Bacterial cultures were diluted in PBS to the cell density corresponding to ~1 × 10^8^ colony-forming units per ml (CFU/mL). These diluted bacterial cultures were inoculated on Mueller–Hinton agar plates (Oxoid, UK), and the prepared samples were firmly applied onto these agar plates. The inhibition zones were measured after incubation for 16 h at 37 °C.

Fluorescent Microscopy. The presence of bacteria (*S. aureus*) after the previous disk diffusion method was checked by microscopy. The collagen scaffolds were placed in 1 mL of PBS and shook thoroughly. The bacteria obtained from the scaffolds were washed with PBS and centrifugation. Fluorescent dye SYTO9 (Thermo Fisher Scientific, Waltham, MA, USA) was used for this assay, and bacterial cells were observed by an Olympus IX71 inverted fluorescence microscope (Olympus, Tokyo, Japan), with an excitation wavelength of 460–495 nm.

Broth Method. The bacteria growth were diluted in Mueller–Hinton broth (Oxoid, UK) to the cell density of ~1 × 10^6^ CFU/mL. Each test tube contained 1 mL of this diluted culture, followed by a piece of the prepared sample. Samples were incubated for 24 h at 37 °C with continuous rotation (Rotator Multi Bio RS-24, Biosan, Latvia), and the optical density (600 nm) was measured at predetermined time intervals.

## 3. Results and Discussion

The critical requirements for the scaffold to be considered as an appropriate material for application in TE are the following: good imitation of the host tissue to provide sufficient biomechanical support for promoting neo-tissue growth, high porosity and water-retaining properties for nutrient delivery, and metabolite removal and a controllable degradation rate. Porous materials made from bovine collagen fulfill these requirements by their structural and chemical integrity closely resembling ECM. Due to the extraction and purification processes, reagent grade bovine collagen lacks parts of its native cross-linked integrity and stability [45]. An additional cross-linking procedure is recommended to improve the structural and biomechanical properties of collagenous material. Cross-linking is used to re-establish multiple inter- and intramolecular cross-links of native collagen structure [46]. Using EDC in the presence of NHS as a cross-linking system has been proven to be one of the most successful chemical cross-linking methods utilized to cross-link the systems with amino and carboxylic groups, where EDC is used as an activator of carboxylic groups and NHS is used as a catalyst improving the cross-linking efficiency [47,48]. As for the cytotoxicity concerns of EDC/NHS cross-linking, in our previous study [49], we proved the nontoxicity of EDC/NHS-stabilized C and CCH scaffolds on 3T3 mouse fibroblasts viability and ex ovo vascularization experiments. Therefore, in this study, a similar EDC/NHS cross-linking protocol was applied.

### 3.1. Microstructure Imaging and Evaluation 

**Figure 1:**
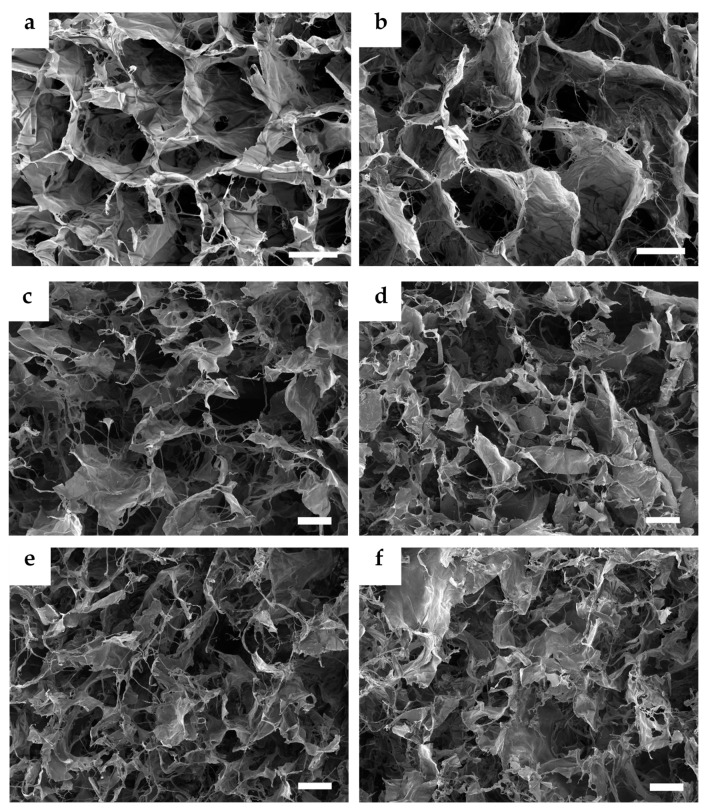
Cross-sectional SEM images of the following samples: C (**a**), CCH (**b**), C/SeCH (**c**), CCH/SeCH (**d**), C/SeCE (**e**), CCH/SeCE (**f**). Images of sectioned porous scaffolds were acquired at 250–274× magnification with a scale of 200 µm, displaying an area of 1.4 × 1.0–2.0 × 1.5 mm^2^.

**Figure 2:**
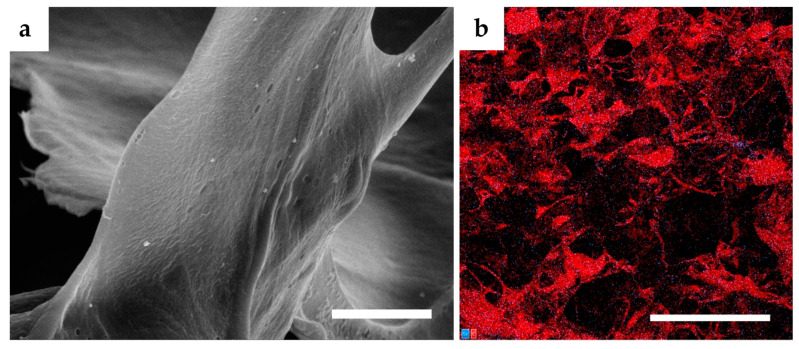
(**a**) SEM image of the C/SeCH scaffold acquired at 25,000× magnification with a scale of 5 µm; (**b**) energy-dispersive X-ray analysis image of the C/SeCH scaffold acquired at 175× magnification with a scale of 500 µm. The red color represents carbon elements and the blue color represents selenium elements.

**Figure 3:**
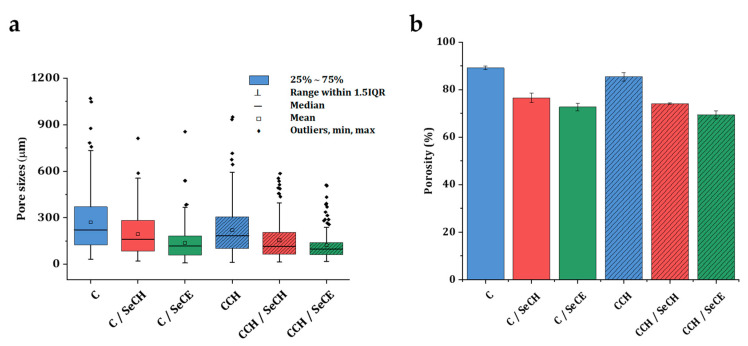
Box plot of C-based and CCH-based scaffolds (**a**) representing a comparison of the pore size distribution, where the box represents the interquartile range (IQR) between 1st and 3rd quartiles. The lines represent the median, statistical minimum, and maximum represented by whiskers as a range within 1.5 IQR and the real minimum and maximum of the dataset included in outliers. Column plot of all types of C-based and CCH-based scaffolds (**b**) representing the percentage porosity of the collagen-based scaffolds enriched with two types of SeNPs.

A fittingly designed pore structure is an essential prerequisite in the development of scaffolds for tissue regeneration. Interconnected pores can positively boost the toleration and the regenerative potential of the material in the wound site. By allowing diffusion of metabolites, oxygen, and growth factors into and out of the material, interconnected pores mediate the cell viability and proliferation [50]. Interconnection of pores goes hand in hand with pore size. Smaller pores 85–120 μm in size may lead to the formation of a cellular capsule around the edges of the scaffold resulting in necrotic regions within the construct. Conversely, larger pores above 325 μm decrease the surface area and limit cell adhesion [51]. Fibroblasts exhibit optimal cell proliferation in porous scaffolds with a pore size of 200–250 μm and a porosity of approximately 86% [52]. The porous structure of the presented scaffolds was achieved by the freeze-drying procedure. Ice crystal formation inside the structure of materials followed by water sublimation fabricated an interconnected porous microstructure inside the scaffolds. Figure 1a,b show the porous morphology of both the C and CCH scaffolds. The so-called honeycomb porous structure can be observed in both the C and CCH scaffolds. The honeycomb-type structure provides the natural ECM environment with the necessary mechanical support and biochemical interplay for cells [53]. The addition of SeNPs visibly changed the porous structure morphology. Biopolymeric substances, which function to stabilize NPs upon agglomeration, partly destroyed the defined porous honeycomb structure (Figure 1c–f). The porous structure of these scaffolds appears to be filled with fibers from chitosan and cellulose that change the finely defined honeycomb structure of pure C and CCH scaffolds. The SeNPs were homogeneously dispersed throughout the structure of the scaffolds (Figure 2). Following the box plot of pore size distributions in Figure 3a, the structure of all the scaffolds was relatively inhomogeneous with outliers of larger pores existing in the structure. The lack of uniformity was predominant in the pure C and CCH scaffolds. The average pore sizes were determined to be 271 µm for the C scaffold and 221 µm for the CCH scaffold with pore sizes ranging between approximately 25 µm and 1 mm for both formerly mentioned scaffolds. Conversely, the addition of biopolymeric suspensions with SeNPs increased the homogeneity of the systems with CCH/SeCE scaffold having the most uniform structure of all the scaffolds. However, with the average pore size of 138 µm for C/SeCE and 123 µm for CCH/SeCE, these scaffolds may not provide a suitable environment for fibroblasts. Scaffolds containing SeNPs bonded to chitosan—C/SeCH and CCH/SeCH—and had average pore sizes of 195 µm and 155 µm, respectively. The trend of pore size distribution followed the trend of porosities determined using absolute ethanol probe. All scaffolds were highly porous, with porosity values ranging from 69% to 89%. The highest porosity was observed in the C scaffold (89%). Similar to the trend of pore size distribution, the addition of chitosan and biopolymer-bonded SeNPs decreased the porosity of the scaffolds. Following the overall results from the morphology and microstructural evaluation, the fabricated collagenous scaffolds alone achieved the most uniform-looking structure with the highest porosity. Polysaccharides added in the form of chitosan or bonded to SeNPs visibly filled the pores and created a more dense structure with lower porosity and pore sizes.

### 3.2. Investigation of Intact Collagen Secondary Structure

**Figure 4:**
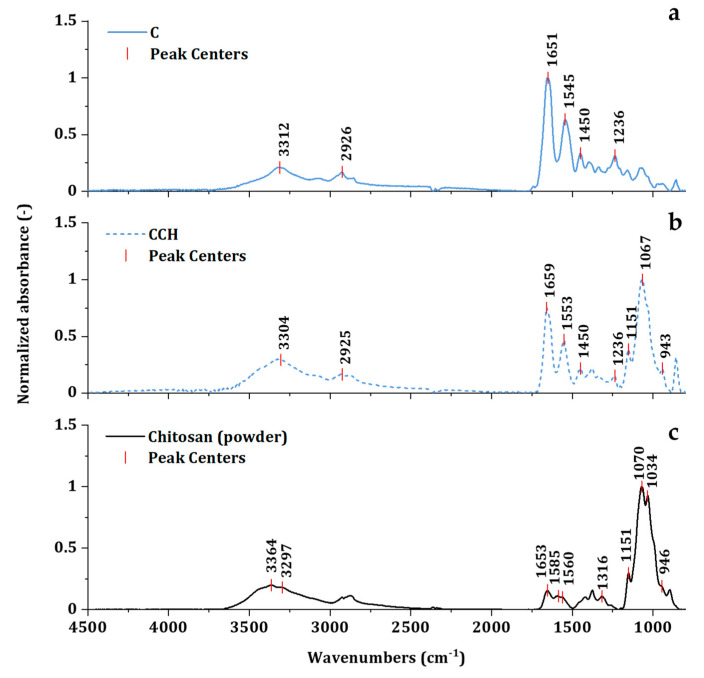
Absorption spectra of C (**a**), and CCH (**b**) scaffolds, and chitosan powder (**c**) with marked peak centers with the appropriate value of wavenumbers.

Collagen stands out above all proteins due to the fact of its biomechanical strength, thermal stability, and biological properties ensuring proper cellular interactions. These remarkable properties are closely connected to the unique secondary triple-helical structure [54]. Evaluation of the collagen secondary triple-helical structure state can be executed by FTIR spectroscopy. In the mid-infrared spectrum of collagen, five characteristic amide bands can be observed—Amide A, B, I, II, and III (Figure 4a). Amide I band, ranging between wavenumbers 1600–1700 cm^−1^, is closely associated with C=O stretching vibration in the acetamide of the amide functional group [55]. Amide III band extending between 1175 and 1310 cm^−1^ is associated with C–N stretching, N–H bending vibrations, C–C stretching, and C–H bending vibrations. Amide II lies in the region of 1500 to 1600 cm^−1^ [56]. The exact positions of all corresponding amide bands, summarized in Table 2, uncovers minor statistically insignificant deviations in positions among all spectra. The addition of chitosan was directly translated to the spectra region below 1200 cm^−1^ (Figure 4b). The band at 943 cm^−1^ displayed the bending of C–H groups in the polysaccharide structure. The vibration determined by the C–O groups in glycosidic bonding can be observed at 1067 cm^−1^. The band at 1151 cm^−1^ corresponded to the asymmetric vibration of C=O of polysaccharide [57]. Corresponding bands can be observed in the pure chitosan spectra at similar or the same positions of 1151, 1070, and 946 cm^−1^ (Figure 4c). The spectra of chitosan register amide bands of residual N-acetylamine groups can be found at 3297 (N–H stretching of Amide A) 1653 (C=O stretching of Amide I), 1516 (N–H bending of Amide II), and 1316 (C–N stretching of Amide III) [58]. These amide vibrations may contribute to minor shifts of amide bands in CCH-based scaffolds. 

From all collagen amide regions, Amide I and Amide III bands corresponded with the secondary structure the most. Amide II reflected conformation changes as well, but it was far less conformation sensitive than the other two regions [56]. The vibration of the Amide I band is directly related to the backbone conformation and hydrogen bonding pattern [43]. Amide III is usually evaluated in correspondence to the band of pyrrolidines (around 1445 cm^−1^). Pyrrolidine functional groups can be found in proline and hydroxyproline amino acids—the main partakers in the stabilization of triple-helix structures through hydrogen bonding [54]. The absorbance ratios of Amide III bands and bands representing pyrrolidines can provide insight into collagen triple-helix integrity [43]. The triple-helix ratio (2) values for denatured collagen are close to 0.5 and those for intact structures are close to 1.0 (Table 2). 

**Figure 5:**
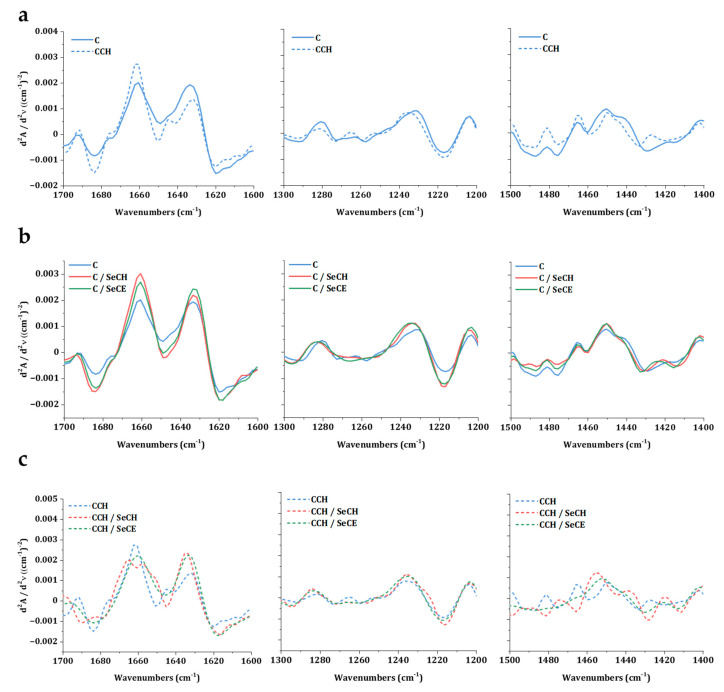
Second derivative spectra of C and CCH scaffolds (**a**), C-based scaffolds with SeNPs (**b**), and CCH-based scaffolds with SeNPs (**c**) exhibiting the Amide I region (1st column), Amide III region (2nd column), and the region of pyrrolidines (3rd column).

The second derivative analysis of Amide I, Amide III and pyrrolidines region revealed more detailed information concerning the structural changes. Comparing the spectra of these regions between C and CCH scaffolds in Figure 5a shows changes in all regions of interest (ROI). It may be assumed, that the cross-linking collagen together with chitosan engaged important sites in collagen structure, that stand behind the formation of intact triple-helix structure. The triple-helix ratio of 0.70 indicated partial denaturation or reorientation of secondary structure in CCH scaffolds. Following the Figure 5c, changes were visible in all CCH-based scaffolds. Lower values of the triple-helix ratio of all CCH-based scaffolds was irregular enough to imply instability of the single secondary structure formation in scaffolds were the chitosan was in situ cross-linked. Interestingly, in the ROI of Amide III, no change was observed. It may suggest that the Amide I region was more sensitive to conformation changes than the Amide III region. The phenomenon of a direct relationship between pyrrolidine and the Amide I ROIs is currently under investigation. No visible changes were registered in C-based scaffolds in Figure 5b as well as no statistically significant deviations were observed between triple-helix ratios of C-based scaffolds. 

### 3.3. Swelling Kinetics

**Figure 6:**
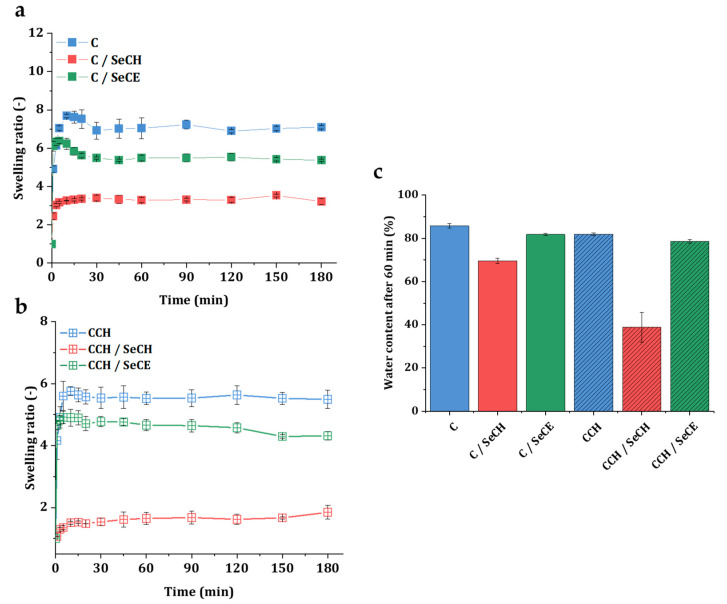
The swelling ratio of the C–SeNPs scaffolds (**a**), CCH–SeNPs (**b**), and water content in the point of equilibrium at 60 min (**c**).

Stability in a water environment and water-retaining properties are essential attributes of regenerative material, since a damp environment can help nutrient transportation and enhance the growth and proliferation of cells [59]. Swelling is intermediated by the hydrophilic character of a C and CCH scaffold’s biopolymeric structure [60]. Figure 6a summarizes the swelling curves of C-based scaffolds and Figure 6b CCH-based scaffolds recorded in 180 min time intervals. All scaffolds presented in this work showed a tendency to swell in a water environment and remained stable during the whole swelling experiment. Overall, in scaffolds with chitosan, either in the form of pure biopolymer or as a SeNP stabilizer, a decrease in the swelling tendency was observed. Compared to other scaffolds, these scaffolds were acting more hydrophobically. The shape of the C/SeCH and CCH/SeCH swelling curves followed slow absorption to the point of absorption equilibrium (after 60 min). In the other four types of scaffolds, higher water absorption was observed in the first 20 min followed by a decrease to the point of absorption equilibrium. Both C and C/SeCE scaffolds showed 10% and 16% growth in the swelling ratio above the absorption equilibrium, whereas CCH and CCH/SeCE scaffolds showed only 4% and 3%growth in the 20 min time interval, respectively. Following Figure 6c, the highest water content at the point of absorption equilibrium was noticed in the pure C scaffold (85%). While CCH, C/SeCE, and CCH/SeCE scaffolds showed high water absorption values of 82%, 82%, and 79%, the addition of SeCH into collagen scaffolds significantly decreased the water absorption down to 70% in the C/SeCH scaffold and 39% in the CCH/SeCH scaffold. The observed swelling phenomenon could be related to the formation of intramolecular ionic bonds between negatively charged collagen and positively charged polysaccharides resulting in a decrease of free hydrophilic functional groups. Although the water content values of the scaffolds containing SeCH NPs were relatively low, all determined values agreed with the ranges of the water content in human skin [61].

### 3.4. Degradation in a Simulated Physiological Environment

**Figure 7:**
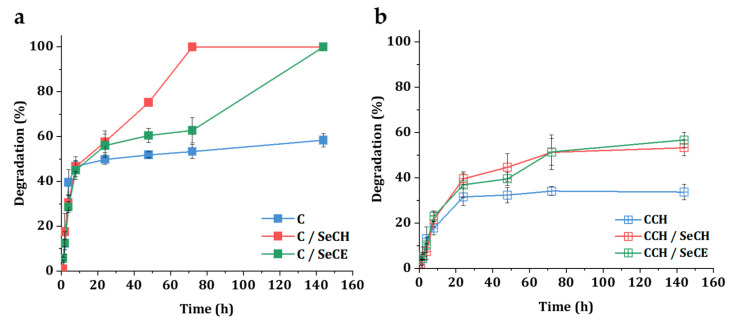
Degradation in the presence of collagenase (in a PBS environment with a pH 7.4 and at 37 °C) of C-based scaffolds (**a**) and CCH-based scaffolds (**b**) with and without SeNPs.

In wound healing, biodegradation of collagenous material is a desired process, since the degradation products induce a chemotactic attraction of human fibroblasts [62]. Moreover, biodegradation kinetics evaluation offers valuable insight into the role of the scaffold in the physiological environment and the understanding of the SeNPs’ release. As a structural protein, collagen is resistant to hydrolysis and in the physiological environment collagen degrades via collagenases—enzymes from a family of Zn^2+^ matrix metalloproteinases (MMPs). Collagen types I–III are cleaved by MMP-1, MMP-2, MMP-8, MMP-13, and MMP-14 [63]. In this work, we simulated degradation in vitro using 0.01 M PBS solution and collagenase enzyme isolated from *Clostridium histolyticum*. Figure 7a summarizes enzymatic degradation of C-based scaffolds with or without SeNPs. Up to 24 h, all C-based scaffolds degraded at a similar pace. After 24 h, both types of SeNP-modified C-based scaffolds continued to degrade up to full disintegration after 144 h, whereas the pure C scaffold lost only 8.6% of its total weight during the same time interval. Faster disintegration of C/SeCH and C/SeCE scaffold indicates the effect of SeNPs on the cleaving activity of collagenase. Though no specific findings on the exact effect of selenium to collagenase activity have been reported to this day, collagenase has been reported to bind other divalent metal ions than Zn^2+^ that can alter the activity of the protein [64]. We suspect that SeNPs may further stabilize the active site of collagenase and increase the rate of enzymatic degradation. The biodegradation of CCH-based scaffolds with or without SeNPs is illustrated in Figure 7b. The existence of chitosan in the structure enhanced the structural stability, increased the materials’ resistance against collagenase, and overall slowed down the biodegradation process, although a faster pace of biodegradation was still observed in SeNP-modified CCH matrices. After 144 h, the CCH/SeCH and CCH/SeCE scaffolds lost 53% and 57% of their total weight, respectively, whereas the pure CCH scaffolds lost 34% of their total weight.

### 3.5. Release Kinetics of SeNPs

**Figure 8:**
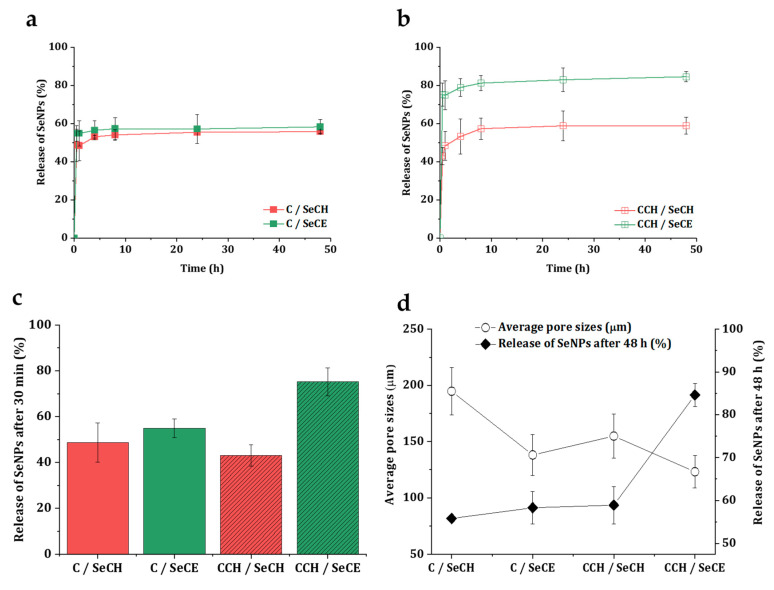
Percentage of release of both types of used SeNPs from the C-based (**a**) and CCH-based (**b**) scaffolds. Summary of the total number of released SeNPs after 30 min (**c**). Comparison of the trends of the average pore sizes with the total number of released SeNPs after 48 h (**d**).

A good understanding of the mechanisms involved in the release of bioactive compounds is important for the fitting design of material with antibacterial properties. The ideal release profile of an antibacterial additive is a combination of rapid initial release and slower, sustained release over a longer period of time [65]. In Figure 8a,b, the release rate of SeNPs from C-based and CCH-based scaffolds is illustrated. An initial burst followed by a slow release of SeNPs could be observed in both C-based and CCH-based matrices. In the 30 min interval, 49% and 55% of the SeNPs were released from the C/SeCH and C/SeCE scaffolds and 43% and 71% from the CCH/SeCH and CCH/SeCE, respectively (Figure 8c). After 8 h, the release profiles became relatively stable and a minimal number of SeNPs were further released. In the time interval between 8 h and 48 h, the scaffolds released only 1.0% to 3.3% SeNPs, where the C/SeCE scaffold released 1.0% and the CCH/SeCE scaffold 3.3%. As observed, a large fragment of the total number of NPs did not release from the scaffolds at all. Possibly, the quick release of SeNPs covered the surfaces of the materials. Non-released SeNPs might be incorporated deeper into the structure of the material and could be released after the start of the disintegration of the material (in the presence of enzymes). This phenomenon is supported by the comparison of total number released after 48 h with the average pore sizes of the scaffolds (Figure 8d). The smaller the pores, the higher the number of released SeNPs, possibly caused by inferior permeation of SeNPs inside the whole structure during the preparation procedure of the scaffolds. Generally, observed kinetics could work well for intended purposes—quick-released SeNPs would successfully inhibit ongoing infection in the wound site and after the disintegration of the materials slowly released SeNPs would safeguard the wound against further infection. 

### 3.6. Antibacterial Testing

**Figure 9:**
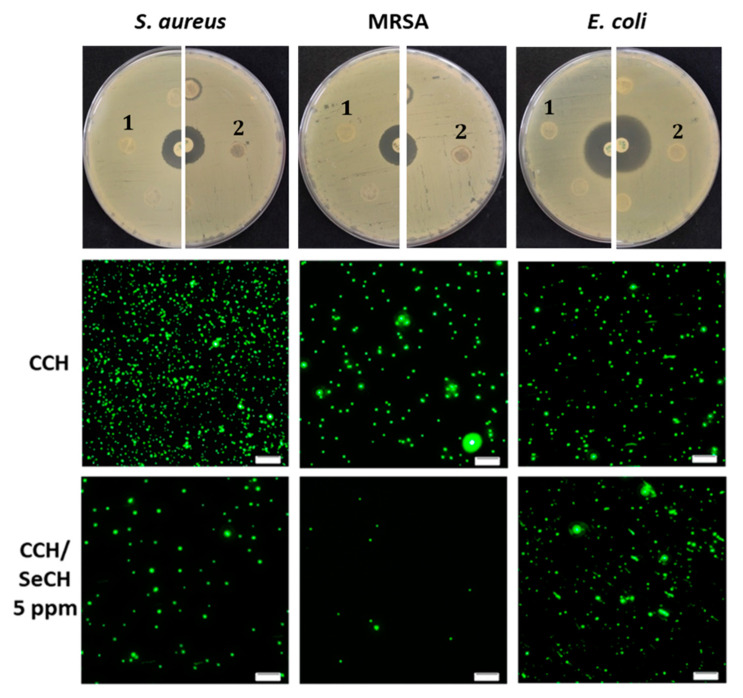
Top row: digital photograph of the disk diffusion method for *Staphylococcus aureus* (*S. aureus*), methicillin-resistant *Staphylococcus aureus* (MRSA) and *Escherichia coli* (*E. coli*) with scaffolds: CCH (1), CCH/SeCH 5 ppm (2), and antibiotic as the positive control (PC). Second and third row: fluorescence microscopy of bacterial cells (SYTO9, green) obtained from scaffolds used in the disk diffusion method; the scale bar is 20 µm.

Due to the interactions and bonding of antibacterial agents into the scaffold structure, it is possible that agents, which normally possess antibacterial properties, can lose their properties after addition into scaffolds, though it is necessary to evaluate bacterial inhibition of prepared materials. For the needs of this study, we tested the antibacterial character of pure collagen scaffolds, collagen–chitosan blends and collagen–chitosan blends containing SeNPs at different concentrations (1, 5 or 10 ppm). For the disk diffusion method, the scaffolds were formed into similar shapes, such as commercial disks, and used the same way. In Figure 9 (top row) the inhibition zones of CCH-based scaffolds with and without SeCH were compared. The CCH/SeCH scaffold with 5 ppm showed small inhibition in the direct surroundings of the disk in presence of both susceptible and antibiotic-resistant strains of *S. aureus*. There were no inhibition zones around the scaffolds with no SeNPs present in the structure. Also, Gram-negative *E. coli* was not inhibited at all. However, some NPs report lower diffusivity through the agar media, and their reduced penetration causes the limitation of diffusion technique [66]. Therefore, for the evaluation of these findings, fluorescence microscopy of bacterial cells obtained from these scaffolds was performed (Figure 9). The membrane permeant fluorescence dye SYTO9 was employed for staining of the nucleic acid of all cells [67]. The fluorescence images of *S. aureus*, MRSA and *E. coli* obtained from the CCH revealed many green spots representing bacteria. When the CCH/SeCH 5 ppm was examined, a considerable reduction of Gram-positive bacteria was shown (Figure 9, bottom row). On the contrary, the growth of *E. coli* was not affected by the presence of scaffolds. These results are in accordance with the determined inhibition zones. The resistance of Gram-negative bacteria to used antimicrobial NPs can be induced by their well-known low permeable outer membrane and also by the presence of efficient efflux system, which pumps out the wide range of antimicrobials from the cell [68].

**Figure 10:**
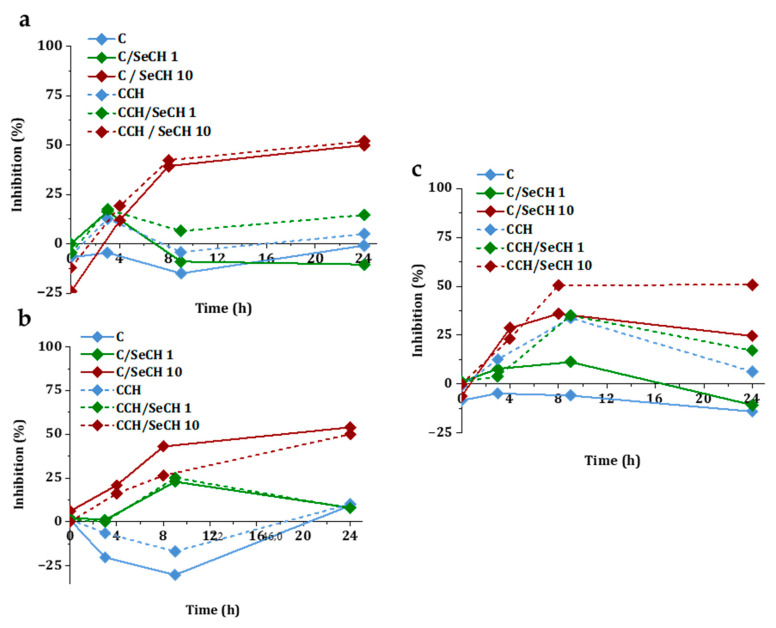
Time-dependent bacterial inhibitory curves for *S. aureus* (**a**), MRSA (**b**), and *E. coli* (**c**). Bacterial samples were incubated in media with pure scaffolds and scaffolds of Se concentrations of 1 ppm or 10 ppm.

The percentage inhibition of bacterial cultures after incubation with scaffolds containing SeNPs at concentrations of 1 ppm and 10 ppm are illustrated in Figure 10. The turbidity was measured at different time intervals for 24 h. The highest inhibition of *S. aureus* (Figure 10a) growth was found for C/SeCH 10 ppm (50%) and CCH/SeCH 10 ppm (52%) after 24 h of incubation. The other scaffolds caused inhibition at most at 17% (CCH/SeCH 1 ppm), whereas scaffolds with chitosan achieved better reductions. Moreover, collagen alone supported the growth of bacteria. After 24 h of incubation, the inhibitory effect was reduced. In the case of MRSA (Figure 10b), the scaffolds with 10 ppm showed a similar efficiency to *S. aureus*, specifically C/SeCH 10 ppm (54%) and CCH/SeCH 10 ppm (50%). Conversely, the scaffolds without SeNPs (C, CCH) increased the growth of MRSA. Also, 10 ppm scaffolds with SeNPs showed inhibition towards *E. coli* (Figure 10c). After 24 h incubation, C/SeCH 10 ppm and CCH/SeCH 10 ppm caused 25% and 51% inhibition, respectively. The C-based scaffolds reported lower inhibitions compared to CCH-based ones. These results differ from the study of Geoffrion and colleagues [69], where the naked SeNPs better reduced the proliferation of Gram-negative bacteria, but the inhibition was dependent on the Se concentration as in our case. On the contrary, the antibacterial activity of SeNPs with different sizes (43–205 nm in diameter) was found for *S. aureus* and MRSA. Moreover, the significant size dependence was identified [70]. 

**Figure 11:**
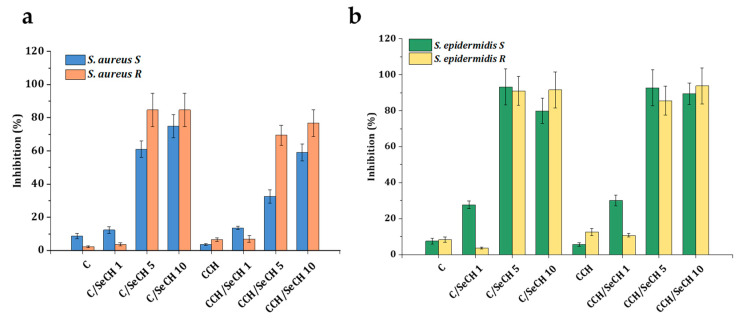
Bacterial growth inhibition of sensitive (S) and resistant (R) clinical isolates: *S. aureus* (**a**) and *S. epidermidis* (**b**). Bacterial samples were incubated for 24 h in media with scaffolds of Se concentrations of 1, 5 or 10 ppm.

For the evaluation of the antibacterial activity of prepared scaffolds for wound treatment application, the bacterial cultures from clinical practice were tested. Among the common pathogens causing skin and soft tissue infections include, for instance, *S. aureus*, *P. aeruginosa*, *E. coli*, and *S. epidermidis* [71]. From the previous tests it is obvious that the scaffolds with SeNPs worked better against Gram-positive bacteria; therefore, for further analyses *S. aureus* and *S. epidermidis* obtained from infected patients wounds were utilized. As one of the major skin pathogens, *S. aureus* can colonize hosts asymptomatically; however, it can also cause a variety of skin infections. Oportunistic pathogenic bacteria *S. epidermidis* is usually beneficial to the host; nevertheless, it can also induce death in premature infants and nosocomial infections [72]. Two clinical isolates from each species were employed for inhibition effect testing: sensitive (S) and resistant (R). The phenotype of both resistant isolates, *S. aureus* and *S. epidermidis*, was confirmed as beta-lactamase producing and methicillin-resistant. The tested Se concentrations were 1, 5, and 10 ppm (Figure 11). The C-based and CCH-based scaffolds as well as samples with 1 ppm Se inhibited *S. aureus* growth (Figure 11a) only up to 14% (CCH/SeCH 1 ppm). The higher Se concentrations (5 ppm and 10 ppm) inhibited the growth of sensitive isolate from 33% to 75%, whereas the highest reduction caused C/SeCH 10 ppm. The resistant clinical strain was reduced more compared to the sensitive one, the 5 ppm and 10 ppm concentrations inhibited the proliferation from 69% to 85%. The 85% inhibition was found for C/SeCH 5 ppm and 10 ppm. The apparent difference between treatment of sensitive and resistant *S. aureus* was observable for scaffolds with 5 ppm SeNPs. The inhibition pattern for *S. epidermidis* was similar to previous observations (Figure 11b). The scaffolds without SeNPs or with 1 ppm of Se reduced the clinical isolate proliferation less than higher SeNPs concentrations. But compared to clinical *S. aureus*, scaffolds with 1 ppm Se decreased the growth two times more (the percentage of inhibition up to 30%) in the case of sensitive *S. epidermidis*. The C-based and CCH-based scaffolds containing 5 ppm and 10 ppm SeCH had a similar reduction effect to both clinical isolates, from 80% to 94% of inhibition. 

## 4. Conclusions

In this study, porous antibacterial collagenous scaffolds enriched with chitosan and SeNPs were investigated for their potential future application in tissue engineering of infected wounds. The high porosity of collagenous scaffolds was achieved by freeze-drying. The porosity was influenced by the presence of antibacterial additives in the matrix. Whereas pure collagen scaffolds exhibited a porosity of 89%, chitosan and SeNPs additives reduced the porosity by 69%. Findings from the second derivative analysis of infrared spectra suggested that cross-linking of chitosan and collagen may alter the collagen triple-helical structure. No alterations were observed in the SeNP-modified collagen-based scaffolds. Water retaining properties of collagenous scaffolds reinforced by EDC/NHS-mediated cross-linking were high (79–85%), whereas the addition of both chitosan and chitosan-stabilized SeNPs into collagenous matrices decreased water absorption by 39%. Degradation in a simulated physiological environment showed increased cleaving ability of collagenase in the environment of scaffolds releasing selenium nanoparticles from their structure. These SeNP-modified collagenous scaffolds disintegrated fully within 144 h. The presence of chitosan in the collagenous matrices significantly slowed down collagenase-mediated degradation. The SeNP-modified collagenous scaffolds at SeNPs concentrations low as 5 ppm showed a strong antibacterial effect (up to 94% of bacterial growth inhibition) towards laboratory and clinical isolates of Gram-positive bacteria from the genus *Staphylococcus.* Based on the presented results, collagenous matrices enriched with chitosan and SeNPs are good candidates for further use in the field of infected tissue regeneration. 

## Figures and Tables

**Table 1:** Matrix composition summary of collagen (C) and chitosan (CCH) scaffolds both pure and modified by selenium nanoparticles with chitosan (SeCH) or selenium nanoparticles with carboxymethyl cellulose (SeCE).

Sample Name	Matrix composition			
	Collagen (%)	Chitosan (%)	SeCH (ppm)	SeCE (ppm)
C	100	-	-	-
C/SeCH	100	-	1, 5, 10	-
C/SeCE	100	-	-	1, 5, 10
CCH	50	50	-	-
CCH/SeCH	50	50	1, 5, 10	-
CCH/SeCE	50	50	-	1, 5, 10

**Table 2:** Characteristic bands and triple-helix evaluation of all C-based and CCH-based scaffolds.

Scaffold	Wavenumbers [cm^−1^]						Triple-Helix Ratio
	Amide A	Amide B	Amide I	Amide II	Amide III	Pyrrolidine	
C	3312	2926	1651	1545	1236	1450	0.94
C/SeCH	3325	2932	1657	1551	1236	1452	0.95
C/SeCE	3319	2930	1657	1551	1238	1452	0.96
CCH	3304	2925	1659	1553	1236	1451	0.70
CCH/SeCH	3325	2928	1655	1551	1236	1454	0.87
CCH/SeCE	3321	2935	1655	1553	1239	1452	0.80
